# Kernohan-Woltman Notch Phenomenon: A Case of Paradoxical Hemiparesis

**DOI:** 10.7759/cureus.76987

**Published:** 2025-01-06

**Authors:** Catarina Negrão, Maria Carolina Silva, Marta Machado, Margarida Mourato, Carolina Chumbo, Ana Rita Tomás, Constança Coutinho, Ana Carolina Monteiro, Clara Matos

**Affiliations:** 1 Department III of Internal Medicine, Hospital Professor Doutor Fernando Fonseca, Amadora, PRT; 2 Department of Nephrology, Hospital Professor Doutor Fernando Fonseca, Amadora, PRT; 3 Department II of Internal Medicine, Hospital Professor Doutor Fernando Fonseca, Amadora, PRT

**Keywords:** false localizing signs, kernohan-woltman notch phenomenon, paradoxical hemiparesis, subdural hematoma, uremic bleeding

## Abstract

The Kernohan-Woltman phenomenon, a rare and often misunderstood neurological condition, is recognized as a false localizing neurological sign. This phenomenon is characterized by a motor deficit ipsilateral to the site of the neurological lesion and occurs due to the mass effect of supratentorial lesions causing a shift of midline structures and subsequent transtentorial herniation, leading to compression of the contralateral corticospinal (pyramidal) fibers within the cerebellum, ipsilateral to the lesion.

This case describes a patient with human immunodeficiency virus-1 and chronic kidney disease who presented to the emergency department with uremic syndrome and a hypertensive crisis. She experienced sudden neurological deterioration, manifesting as prostration, anisocoria, and left hemiparesis, in the context of an acute subdural hematoma of the left cerebral hemisphere, which is a rare complication of hypertensive crisis. The fact that the hemiparesis was on the same side as the hematoma indicates a rare neurological sign, known as the Kernohan-Woltman phenomenon.

This case is particularly noteworthy due to the rare coexistence of these conditions and the atypical presentation of neurological symptoms in the context of supratentorial lesions. The case emphasizes the importance of recognizing this phenomenon in patients with mass-effect lesions, especially in the setting of critical illness, as early identification can influence clinical decision-making and management strategies. Given the rarity of this phenomenon, this report contributes to the literature by providing insight into its clinical presentation and diagnostic challenges, highlighting its relevance in neurocritical care.

## Introduction

The Kernohan-Woltman Notch Phenomenon (KWNP) is defined as a compression of the contralateral cerebral peduncle against the tentorial notch, and it is one of the most widely known pathophysiological mechanisms causing paradoxically ipsilateral hemiparesis and mydriasis [1,2]. This phenomenon can occur in patients with brain tumors and severe head injuries, such as subdural hematoma (SDH)[1].

Chronic kidney disease (CKD) patients experience multiple well-known complications, including an increased risk of bleeding, primarily associated with platelet dysfunction and anemia [3]. One of the most common hemorrhagic manifestations described in uremic syndrome is SDH [4]. Although the pathophysiology of SDH has yet to be fully elucidated, it is believed to be multifactorial. Acute spontaneous SDH, though rare, can also occur as a form of neurological end-organ damage during hypertensive crises [5]. This condition is considered a hypertensive emergency and requires prompt and rigorous blood pressure management to prevent further complications.

We describe a case of KWNP associated with a spontaneous acute SDH with paradoxical presentation of ipsilateral weakness and anisocoria in a young woman with CKD, who presented to the emergency room with a hypertensive emergency and uremic syndrome.

## Case presentation

A 42-year-old female of African descent, originally from Guinea-Bissau and residing in Portugal for the past decade, presented to our emergency department with symptoms of asthenia, fatigue, nonspecific malaise, nausea, and vomiting. She had no history of trauma, fever, peripheral edema, chest pain, or dyspnea.

The patient had a history of irregular medical follow-up for human immunodeficiency virus-1 (HIV-1) infection treated with abacavir, lamivudine, and dolutegravir, CKD stage 5 due to focal segmental glomerulosclerosis in the pre-hemodialysis phase, hypertension, and Wolff-Parkinson-White syndrome.

Upon admission, she was hypertensive (systolic blood pressure ~190-200 mmHg), tachypneic, alert, and oriented but lethargic with sparse speech without focal neurological deficits. Arterial blood gas analysis revealed metabolic acidosis (pH 7.15, pCO_2_ 20.8 mmHg, pO_2_ 116 mmHg, HCO_3_ 7.1 mmol/L, lactate 1.1 mmol/L). Blood tests revealed anemia (hemoglobin 5.6 g/L) with no other hematologic abnormalities, worsening renal function (creatinine 36.9 mg/dL, urea 395 mg/dL, eGFR 1 mL/min./1.73 m^2^) without hyperkalemia, hypocalcemia (ionized calcium 0.93 mmol/L), with normal lactate dehydrogenase and haptoglobin levels.

In the presence of severe metabolic acidosis, elevated creatinine and urea, and lethargy, a diagnosis of acute-on-CKD with uremic syndrome was considered, and the patient was started on fluid therapy and bicarbonate while awaiting emergent hemodialysis initiation. Due to hypertension, she received 3 mg of isosorbide dinitrate, with no response. She then experienced a sudden neurological decline, with a Glasgow Coma Scale (GCS) score of 9 (Eye 2, Verbal 2, Motor 5), severe dysarthria, anisocoria (left-sided mydriasis), leftward gaze deviation, left hemianopsia, left-sided hemihypoesthesia, and grade 2 left-sided hemiparesis. A cerebral computed tomography (CT) scan revealed a left hemispheric SDH with significant midline shift and uncal herniation, with no abnormalities on cerebral angiography.

**Figure 1:**
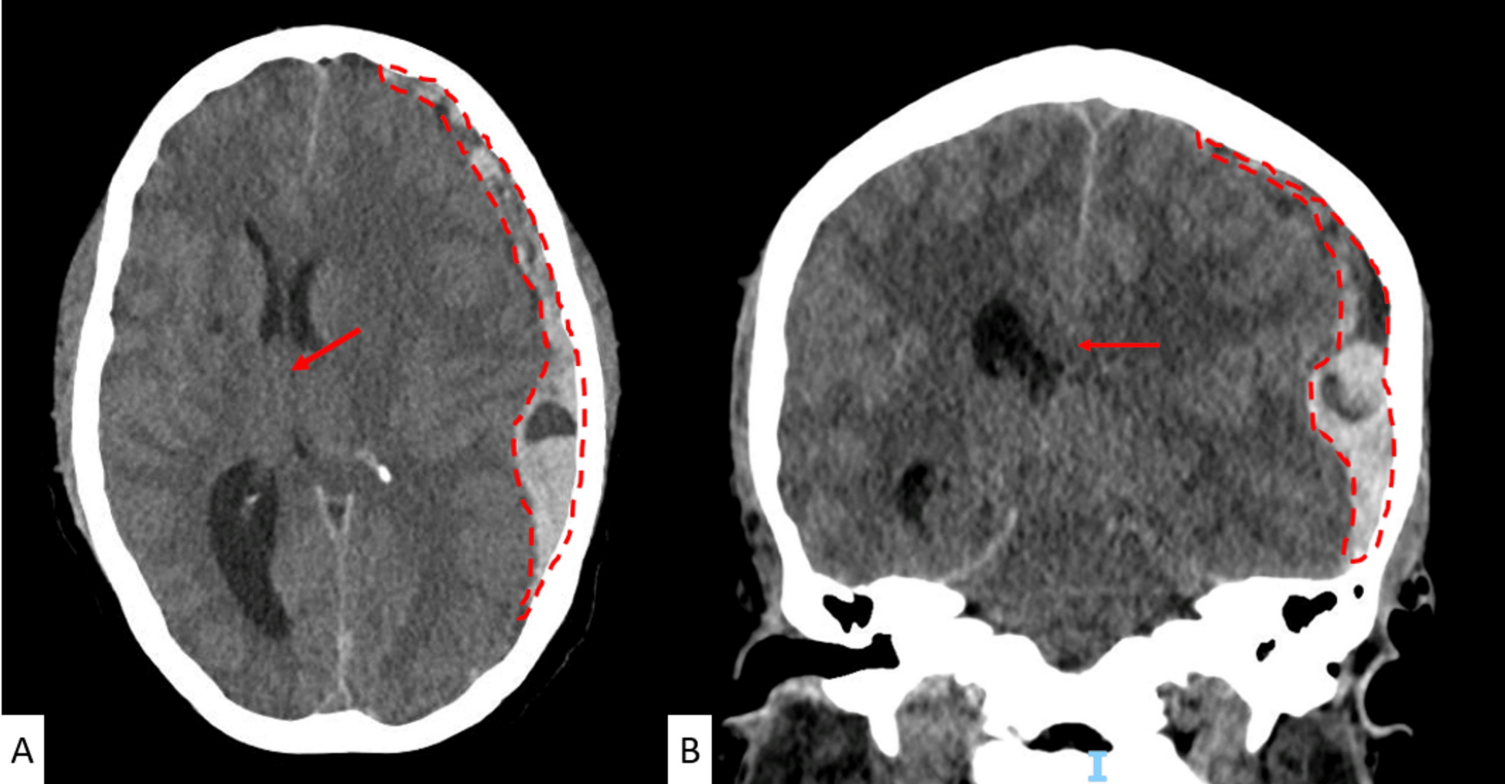
CT scan of the cranium (axial view (A) and coronal view (B)) with evidence of a subdural hematoma in the left hemisphere (red dashed line) with significant midline shift (red arrow) and uncal herniation.

Given these findings, she underwent orotracheal intubation for airway protection and was started on urapidil infusion for blood pressure control. She was transferred to a Neurosurgical Intensive Care Unit for decompressive craniectomy. Her hospitalization was prolonged, requiring induction of dialysis and management of a respiratory infection. However, she was eventually discharged with significant neurological improvement, persisting only a grade 3 left-sided hemiparesis.

## Discussion

KWNP is a rare and complex clinical and radiologic condition typically associated with significant side-to-side brain shift following acute temporal mass effects. Although it is recognized in the medical field, KWNP remains incompletely understood [1,2].

This phenomenon was first described by Kernohan and Woltman in 1929 during a post-mortem examination of a brain tumor patient who had a notched cerebral peduncle due to contralateral herniation [6,7]. Since then, KWNP has been associated with conditions like brain tumors and supratentorial hematomas. KWNP is more commonly described in association with traumatic events, although it can also occur due to spontaneous disease processes [1].

KWNP is considered an exceptionally rare phenomenon, according to the most recent literature reviews, which mainly consist of case reports. However, its incidence might actually be underestimated [1,7].

KWNP occurs when an intracerebral lesion causes uncal herniation, which in turn leads to a substantial displacement of the brainstem. This displacement compresses the contralateral cerebral peduncle against the free edge of the cerebellar tentorium. Over time, the compression associated with KWNP can compromise the integrity of the crus cerebri (the frontal part of the cerebral peduncle) and the descending corticospinal tract, resulting in a range of neurological deficits [1,2,8]. The most frequently observed symptoms include ipsilateral motor deficits, a reduced level of consciousness, and pupil abnormalities, with ipsilateral mydriasis being the most commonly reported [1].

The diagnosis of KWNP relies on a combination of neurological examination and neuroimaging. While CT scans can identify the mass effect of a lesion compressing the brainstem, a magnetic resonance imaging (MRI) scan is more effective in detecting brainstem abnormalities, such as deformities in the cerebral peduncle caused by transtentorial herniation. The phenomenon is typically observed in coronal T2-weighted and FLAIR MRI sequences, where it presents with a characteristic peripheral triangular shape. Additionally, MRI often shows a small hypointense area on T1-weighted images and a hyperintense area on T2-weighted images. Diffusion tensor imaging can further reveal disruptions in the affected cerebral peduncle [9].

In this case, only a single CT scan was conducted in the emergency room due to the patient’s clinical condition. However, the neurological findings were consistent with the KWNP.

Renal insufficiency increases the risk of bleeding, which is linked to platelet dysfunction and imbalances in endothelial function [3,10]. This bleeding risk is evident in patients with CKD, whether they are on dialysis or not, as well as in those with acute kidney injury. Contributing factors include systemic coagulopathy, the effects of hemodialysis and anticoagulation, and underlying conditions like lupus and thrombotic thrombocytopenic purpura [4]. Anemia associated with CKD further worsens bleeding risk by reducing platelet aggregation and interaction with blood vessel walls [11,12].

SDH are among the most commonly observed hemorrhagic complications in patients with CKD. Individuals with moderate to severe CKD who suffer intracranial bleeding often have larger hematomas and a greater incidence of intraventricular hemorrhage compared to those with normal renal function [4]. Moreover, these patients are more likely to experience hemorrhages in the lobar regions of the brain, rather than in the deep basal ganglia, brainstem, or cerebellum, which are typically affected by hypertensive bleeds [13].

The management of a life-threatening SDH in a patient with uremic syndrome is challenging. In addition to anti-edema measures, emergent decompressive craniectomy is often necessary [1]. Interestingly, aggressively lowering blood pressure can be detrimental in patients with renal impairment and intracranial hemorrhage [14]. Therefore, it is important to reduce blood pressure gradually and avoid significant fluctuations to maintain stable perfusion pressure in these patients. Although clear evidence for specific hemostatic control strategies is lacking, options such as desmopressin, platelet transfusions, and cryoprecipitate may be considered in high-risk situations [13,15].

In this case, we describe a patient who experienced an acute-on-CKD with uremic syndrome. She also had anemia and poorly controlled hypertension, which resulted in an acute spontaneous SDH. This condition was characterized by a decreased level of consciousness, mydriasis, and hemiparesis on the side of the hematoma, as shown on the CT scan. These manifestations are consistent with the KWNP.

## Conclusions

This case highlights not only a severe complication associated with uremic syndrome but also a rare clinical presentation. The hemorrhagic diathesis seen in uremic syndrome, due to platelet dysfunction and exacerbated by low hematocrit, as observed in this patient, can manifest as spontaneous SDH. While hypertensive emergencies alone are rarely linked to isolated SDH, in this case, it may have been a contributing factor, particularly given the increased hemorrhagic risk in uremic syndrome.

The clinical presentation of ipsilateral hemiparesis relative to the neurological lesion in a patient with a large SDH was indicative of the KWNP. This rare false localizing sign might induce a misdiagnosis of the location of a cerebral lesion, imposing a radiological diagnostic confirmation to guide an appropriate therapeutic approach.
